# Cycling in the Absence of Task-Related Feedback: Effects on Pacing and Performance

**DOI:** 10.3389/fphys.2016.00348

**Published:** 2016-08-10

**Authors:** Benjamin L. M. Smits, Remco C. J. Polman, Bert Otten, Gert-Jan Pepping, Florentina J. Hettinga

**Affiliations:** ^1^Center for Human Movement Sciences, University Medical Center Groningen, University of GroningenGroningen, Netherlands; ^2^Institute of Sport, Exercise and Active Living, Victoria UniversityMelbourne, VIC, Australia; ^3^Centre of Sport and Exercise Science, School of Biological Sciences, University of EssexColchester, UK; ^4^Department of Psychology, Bournemouth UniversityBournemouth, UK; ^5^Faculty of Health Sciences, School of Exercise Science, Australian Catholic UniversityBrisbane, QLD, Australia

**Keywords:** energy regulation, external device, information, end spurt, race strategy, time trial

## Abstract

**Introduction:** To achieve personal goals in exercise task completion, exercisers have to regulate, distribute, and manage their effort. In endurance sports, it has become very commonplace for athletes to consult task-related feedback on external devices to do so. The aim of the present study was to explore the importance of the presence of this information by examining the influence of the absence of commonly available task-related feedback on effort distribution and performance in experienced endurance athletes.

**Methods:** A 20-km cycling time trial was performed. Twenty Participants from a homogenous cyclist population were appointed to a group that did not receive any feedback (NoF), or a group that could consult task-related feedback (i.e., speed, heart rate, power output, cadence, elapsed time, and elapsed distance) continuously during their trial (FF).

**Results:** The distribution of power output (PO) differed between groups. Most evident is the spurt at the end of the trial of FF, which was not incorporated by NoF. Nevertheless, no between-group differences were found in performance time (FF: 28.86 ± 3.68 vs. NoF: 30.95 ± 2.77 min) and mean PO controlled by body mass (FF: 3.61 ± 0.60 vs. NoF: 3.43 ± 0.38 W/kg). Also, no differences in rating of perceived exertion scores were found.

**Conclusion:** The current study provides a first indication that prior knowledge of task demands together with reliance on bodily and environmental information can be sufficient for experienced athletes to come to comparable time trial performances. This questions the necessity of the presence of in-race instantaneous task-related feedback via external devices for maximizing performance. Moreover, it seems that different pacing strategies emerge depending on sources of information available to experienced athletes.

## Introduction

Athletes are continuously required to make decisions whether to persist in a given behavior or switch to a different one, balancing performance goals against threats of premature exhaustion. Such a dilemma is not limited to the sport context. Engagement in physical activity and a healthy lifestyle requires the selection of appropriate and comfortable intensities for a particular duration to stay sufficiently active. The goal-directed distribution and management of effort across the duration of an exercise bout is also known as *pacing* (Edwards and Polman, 2012). There is an ongoing debate about what influences the selection of an optimal pacing strategy (Smits et al., 2014) or why individuals select a strategy that is too intense, causing premature fatigue, or too conservative, resulting in poor performance or lack of physiological adaptations (Renfree et al., 2014). In view of improving the current understanding of the factors relevant in determining effort distribution in ongoing exercise, the current study considered the importance of commonly available task-related feedback for decision-making in pacing in endurance cyclists.

Pacing and performance can only be optimized if athletes make decisions based on the most relevant information (Renfree et al., 2014). A recent review (Smits et al., 2014) initiated a framework in which pacing is considered as a continuous decision-making process, fuelled by reciprocal interactions between processes internal to the athlete and the environment in which the athlete acts. In addition, it was suggested that the use of bodily and environmental information should not be considered in isolation for a given moment, but also in anticipation to factors such as knowledge of the likely demands of the remaining exercise bout (e.g., certainty about the endpoint and duration) and personal goals (Smits et al., 2014). Moreover, prior experience has been indicated to be important in successfully completing pacing tasks (Mauger et al., 2009; Micklewright et al., 2010; Edwards and Polman, 2013; Smits et al., 2014).

In endurance sports, it has become commonplace for athletes to consult task-related feedback (e.g., current speed, cadence, heart rate, power output, elapsed time and elapsed distance) on external devices. The contribution of such feedback has been critically examined in existing research in the area of deception and pacing strategies (Jones et al., 2013). Research with deceptive feedback-interventions during endurance trials has indicated that (a) pacing strategy selection is based on the perceived distance of a time trial rather than the actual distance (Nikolopoulos et al., 2001); (b) athletes deceived of the actual distance completed the subsequent performance trial based on perceived effort rather than on actual distance (Paterson and Marino, 2004); (c) pacing is influenced by an interaction between feedback and previous experience (Micklewright et al., 2010); and (d) time trial performance does not differ between accurate and inaccurate split-time feedback conditions (Wilson et al., 2012).

Non-deceptive feedback studies have also considered the relation between task-related feedback and pacing. No performance differences were found between groups of inexperienced participants that either did or did not receive prior knowledge of distance and distance feedback during 4-km cycling time trials. It was suggested that the inexperienced participants who did receive task-related feedback demonstrated a greater reliance on afferent feedback (e.g., from heart, lungs, skeletal muscles) than on task-related feedback, and were conservative when setting a pacing strategy (Williams et al., 2012). Other research (Foster et al., 2009) found cautiousness during early trials within unexperienced but fit participants, followed by progressively increased effort during later trials as participants became more confident that the time trial could be completed without unreasonable levels of exertion. It was stated that this cautiousness is not unlike the slower speed of completion that is typically observed in motor learning tasks adopted to reduce errors. A study in which groups of experienced participants did or did not receive prior knowledge of distance and distance feedback during 4-km cycling time trials found better initial trial performance within the group that received feedback (Mauger et al., 2009). This indicates that athletes may choose to pace themselves according to task-related feedback if their experience supports this as a successful strategy (Micklewright et al., 2010). Finally, it has been suggested that it is not the task-related feedback itself that is important, but how an athlete interprets and acts upon it (Micklewright et al., 2010). For example, athletes decided to start an end spurt when they believed that an exercise task is 90% completed (Catalano, 1973).

If pacing is considered as a buffering mechanism to enable successful completion of certain strenuous tasks, then prior experience and accurate knowledge of the task demands are crucial to success (Edwards and Polman, 2013). When we consider prior experience in pacing as familiarity with interpreting and acting upon instantaneous bodily and environmental information in anticipation to likely demands of the remaining task and personal goals, it can be hypothesized that athletes who have gained such experience actually do not need task-related feedback from external devices to successfully complete a task of which the demands are known; even though the task as such might be rather novel, such as cycling a road cycling time-trial. No endurance exercise studies have been found focussing on the necessity of the presence of in-race instantaneous task-related feedback that is nowadays commonly available via external devices (e.g., bike computer, running watch). Therefore, the aim of the present study was to examine the influence of an absence of commonly available task-related feedback on effort distribution and performance in experienced endurance athletes while riding a time trial. To do so, pacing (i.e., power-distribution) and performance during a 20-km cycling time trial of a group that did not receive any instantaneous task-related feedback (NoF) was compared with a group that could consult task-related feedback continuously during the trial (FF). Based upon the above, we expected no inferior performance in NoF compared to FF.

## Materials and methods

### Participants

A homogenous group of 20 experienced and trained [i.e., “performance level 3” (De Pauw et al., 2013)] male cyclists/triathletes (6.4 ± 5.5 years of experience in their sports and 4.6 ± 2.4 training bouts per week), familiar with the process of pacing in their sports, was selected and completed the Physical Activity Readiness Questionnaire (Thomas et al., 1992) and provided written informed consent. The study was approved by a local Ethics Committee and conformed with the Declaration of Helsinki.

### Research design

All participants completed an incremental cycling exercise test (*ICET*) to volitional exhaustion to determine maximal cardiorespiratory values. Furthermore, each participant performed a 20-km cycling time trial as fast as possible while being randomly allocated to an experimental group that received no feedback (NoF) or a control group that was allowed full feedback (FF). Participants did not perform a familiarization trial, as we were interested in imposing a relatively novel task such as cyclists in the Grand Tours are experiencing: each time trial or stage is different, cycled under different conditions. Imposing a familiarized time trial condition in a repeated measures design—instead of a rather novel task in our current design—would compromise ecological validity of the study when interested in road cycling. In addition, we expected that the importance of feedback would be higher in a rather novel task.

All tests were performed in a laboratory with conditioned temperature and relative humidity.

### Incremental cycling exercise test (ICET)

The ICET was performed on a cycle ergometer (Lode Excalibur; Lode BV, Groningen) at a pedal frequency of 80 rpm. After a 10 min warming-up at a work rate of 150 W and 1 min passive rest, the test started on an exercise intensity which was equivalent to 3 W/kg^*^[participant's body mass, kg]. This equivalent provided comparable relative starting exercise intensities for all participants and corresponded to a power output (PO) that would elicit ~65–70% of maximal oxygen consumption (VO_2max_; Hawley and Noakes, 1992; Rønnestad et al., 2011). PO was increased every 2 min by 30 W until the participant reached volitional exhaustion (i.e., cadence < 80 rpm). PO, heart rate (HR), Rating of Perceived Exertion [RPE; Category Ratio version ranged from 0 to 10 (Borg, 1982)], rate of oxygen consumption, and carbon dioxide production were recorded for further analysis. Respiratory gas exchange was measured breath-by-breath using open-circuit spirometry (Oxycon Delta; Enrich Jaeger, Hoechberg, Germany). Before each test, the gas analyser was calibrated using a Jaeger 3-L syringe, room air, and a standard gas mixture (5.04% CO_2_). HR was recorded every 2 s (Polar Electro, Kempele, Finland).

### Time trial

Participants conducted the trial using their own bike mounted on an ergotrainer (Tacx Flow T1680, Wassenaar, The Netherlands). A power meter (CycleOps PowerTap Elite+, Madisson, USA; sample frequency: 1 Hz, accuracy: ±1.5%) was used to record PO, time and covered distance during each trial for subsequent data-analysis. Previous research has shown that this power meter provides valid and reliable PO measurements in laboratory tests (PO range: 100–450 W; Bertucci et al., 2005). Also, participants were asked to rate their perceived exertion (RPE) at least once within every 4-km block, but at irregular intervals (i.e., after 4, 6, 11, 15, 18, and 20-km of the trial completed for the participants in both groups) to avoid that it would provide the feedback-blinded participants any distance or time feedback indirectly. It should be noted that, because the Tacx does not incorporate the non-linear relation between PO and velocity, 20-km cycling on a Tacx is not fully identical to 20-km on the road outside or, for example, on a Velotron ergometer.

### Full feedback (FF) control-group and No Feedback (NoF) experimental-group

For participants allocated to FF (*n* = 10), task-related feedback was provided during the entire trial. As a result, they could continuously consult their PO, speed, HR, cadence, covered distance, and time elapsed. Participants appointed to NoF (*n* = 10) did not receive any feedback during the trial (“blinded”). They only knew they had to cycle 20-km as fast as possible and a stop-sign would be provided when they covered this distance.

Within this experimental design the performance-environment (i.e., exercising in the laboratory) and -goal (i.e., completing the trial as fast as possible) were the same for both groups. However, whereas NoF-participants were reliant on their own resources (i.e., perceived bodily exertion and prior experience with performing time trials) during their trial, participants within FF were able to evaluate their perceived bodily exertion, interim performance, and future task demands via external devices.

### Preparing data for analysis

To examine the pacing strategy and performance of both groups over the trial, participants' PO-distribution curves were considered. In order to compare the PO-distribution between FF and NoF, the mean PO-distribution curves of both groups over the entire trial were established. To do so, first we normalized the PO-distribution curve of each participant to 1250 data points. This number of data points was based on the completion time in seconds of the fastest participant. Following this, the power data was controlled for body mass differences between participants [i.e., participants' PO throughout the trial divided by their body mass (PO, W/kg)]. In addition to considering PO-data (i.e., PO), we were also interested in how the groups relatively distributed their PO over the trial and how the groups' PO was related to the maximal PO-capacity of the participants within the groups. As a consequence, participants' PO throughout the trial was divided by their mean PO over the trial [PO_rel_, –], as well as divided by their peak PO established during ICET [PO_ICET_, –].

To compare overall performance between FF and NoF, calculated group-means of PO and PO_ICET_, and of the performance time [PT] were used. Furthermore, to consider whether there were differences in PO between and within groups at different intervals within the trial, the PO- and PO_rel_-distributions were divided into 10 equal-sized segments (from now on to be called *10%-segments* and abbreviated with *S1* till *S10*, whereas S1 = 0–10%; S2 = 10–20%; etc.). Also, paired differences between neighboring 10%-segments (from now on to be called *change-segment* and abbreviated with *CS1* till *CS9*) were calculated (i.e., CS1 = S2–S1; CS2 = S3–S2; etc.) to examine whether PO-changes over subsequent 10%-segments within the groups differ between the groups. Finally, to consider whether RPE differed between groups, RPE group means were calculated for each time the participants rated their perceived exertion during the trial.

### Analysis

To determine whether there were between-group differences in anthropometric characteristics, and ICET- and overall-performance measures, independent *t*-tests were conducted. Repeated measures ANOVA's were used to examine the effects of feedback condition on PO at different parts during the race (i.e., 10%-segments) and PO-changes over the race (i.e., change-segments). If a main effect for group was found, Bonferroni corrected independent *t*-tests were performed to consider within which specific segment(s) PO differed between groups. If a main effect for segment was found, Bonferroni corrected paired-samples *t*-tests were performed to consider which specific neighboring 10%-segments of PO differed from each other within groups.

Finally, to consider differences in perceived exertion between groups, independent *t*-tests on mean RPE-scores were performed. As RPE was asked at irregular intervals, no repeated measures ANOVA was applied for the RPE-scores analysis.

Effect sizes were calculated as appropriate. An effect size of 0.2 is considered as small, 0.5 as medium, and >0.8 as large (Cohen, 1992). For all tests a two tailed significance was used with an alpha of 0.05.

## Results

### Participants

The group characteristics are provided in Table 1. No between-group differences were found in anthropometric characteristics and cardiorespiratory values.

**Table 1:** **Comparison of anthropometric characteristics and ICET-measures [Mean (*SD*)] of 20 male endurance athletes divided into two groups**.

	**FF^a^**	**NoF^a^**	***p*-value**	***d***	***r***
Age (years) at first test^b^	28.2 (7.8)	27.2 (5.4)	0.91	–	0.034
Height (cm)	186 (5)	188 (6)	0.28	0.50	–
Body mass (kg) at ICET^c^	78.7 (7.9)	76.1 (10.4)	0.54	0.28	–
HR_max_ (bpm)^d^	196 (10)	194 (7)	0.66	0.20	–
PPO (W)^e^	387 (50)	381 (33)	0.73	0.14	–
PPO (W/kg)^e^	4.95 (0.67)	5.04 (0.46)	0.72	0.16	–
VO_2max_ (ml·min^−1^)^f^	4220 (685)	4473 (576)	0.40	0.40	–
VO_2max_ (ml·kg^−1^·min^−1^)^b^, ^f^	53.7 (7.1)	59.0 (7.7)	0.095	–	0.38

a FF, Full Feedback control-group (n = 10); NoF, No Feedback experimental-group (n = 10);

b For the variables which violated assumptions of normal distribution, Mann-Whitney U-Tests were used;

c ICET, incremental cycling exercise test;

d HR_max_, maximal heart rate;

e PPO, peak power output;

f *VO_2max_, maximal oxygen consumption; because of an abnormal result in the VO_2max_-result of one of the participants in NoF, this result has been excluded. Therefore, n_NoF_ = 9 for VO_2max_ (ml·min^−1^) and VO_2max_ (ml·kg^−1^·min^−1^). No differences were found*.

### Overall performance

**Figure 1:**
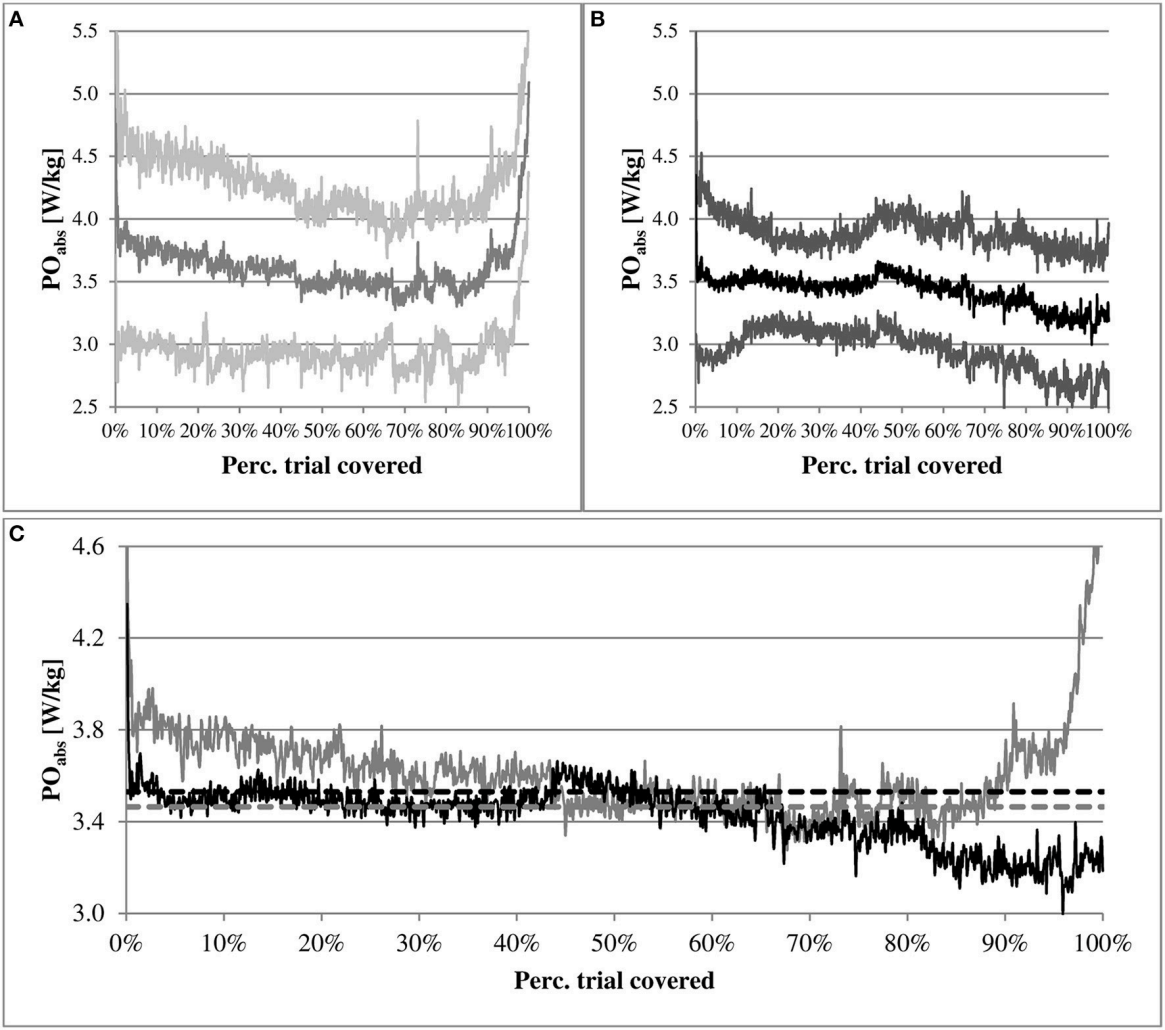
**Mean distribution curves per group of participants' power output divided by their body mass (PO)**. On the top left **(A)** the curve of the Full Feedback (FF) group, and on the top right **(B)** the curve of the No Feedback (NoF) group. The brighter upper and lower curves within both top graphs represent the standard deviations. On the bottom **(C)** the curves of FF (gray) and NoF (black) together. The bottom graph also includes two dotted straight lines that represent boundaries corresponding with 70% of the peak PO established during the incremental cycling exercise test of FF (gray) and NoF (black).

Figure 1 illustrates the mean PO-distribution curves over the entire trial per group (FF top left and NoF top right) and for both groups together (bottom). To visualize how PO over the trial is related to the peak PO established during ICET (PPO), a 70%^*^PPO-boundary per group (dotted lines) is incorporated. The mean PO-distribution curve of FF is usually above or at the 70%^*^PPO-boundary, whereas the curve of NoF is usually situated at or below the boundary. Nevertheless, the higher mean PO_ICET_ in FF [0.73 ± 0.06 (–)], compared to NoF [0.68 ± 0.06 (–)], was not significant, but accompanied by a large effect size (Cohen's *d* = 0.85). Also, differences in mean PT (FF: 28.86 ± 3.68 vs. NoF: 30.95 ± 2.77 min; Cohen's *d* = 0.64) and mean PO (FF: 3.61 ± 0.60 vs. NoF: 3.43 ± 0.38 W/kg; Cohen's *d* = 0.37) between groups were not significant, which indicates an absence of performance differences between groups.

### Segment performance within groups

**Figure 2:**
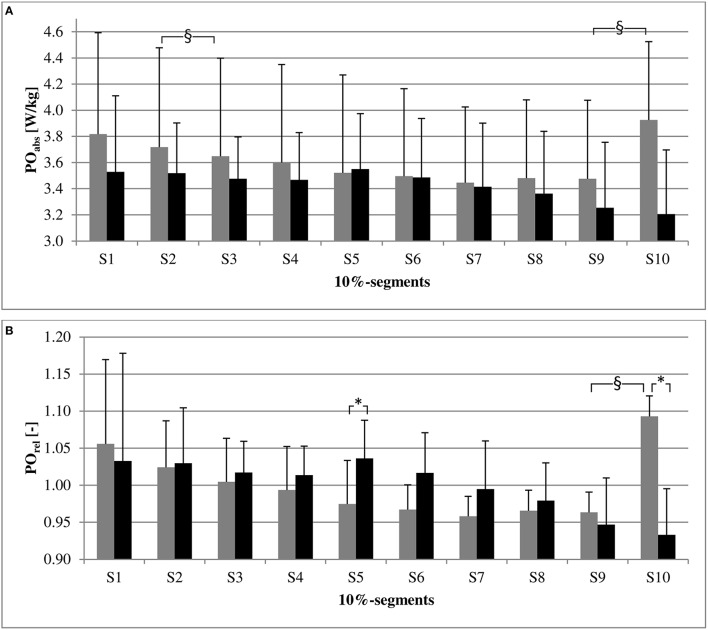
**Comparison of power output (PO) characteristics [Mean (*SD*)] of 10%-segments between and within groups (*n* = 10 per group) for PO (top graph, A) and PO_rel_ (bottom graph, B)**. PO, Mean of participants' power output (PO) divided by their body mass; PO_rel_, Mean of participants' PO divided by their mean PO over the trial; Gray bars, Full Feedback group; Black bars, No Feedback group; 10%-segments, the PO- and PO_rel_-distributions were divided into 10 equal-sized segments (S1 = 0–10%; S2 = 10–20%; etc.). Significant between group differences are marked by ^*^ and within group differences by §.

Figure 2 shows the 10%-segments for both PO and PO_rel_ per group. A segment main effect was found for both PO and PO_rel_ within FF [respectively *F*_(1.66)_ = 5.12; *P* = 0.02, and *F*_(1.70)_ = 4.89; *P* = 0.03]. *Post-hoc* comparisons revealed that mean PO in FF was higher in S10, compared to S9, for both PO [*t*_(9)_ = −5.97, *P* < 0.001; Cohen's *d* = 0.77] and PO_rel_ [*t*_(9)_ = −6.07; *P* < 0.001; Cohen's *d* = 1.87], whereas mean PO in S3 was lower than in S2 for PO [*t*_(9)_ = 3.96; *P* = 0.003; Cohen's *d* = 0.09] and nearly for PO_rel_ [*t*_(9)_ = 3.68; *P* = 0.005; Cohen's *d* = 0.32]. There was no significant main effect for NoF.

### Segment performance between groups

A group by segment interaction effect was found for both PO [*F*_(1.74)_ = 3.97; *P* = 0.03] and PO_rel_ [*F*_(1.77)_ = 3.95; *P* = 0.03]. *Post-hoc* comparisons revealed that mean PO in S10 was higher in FF, compared to NoF, for PO_rel_ [*t*_(18)_ = 4.94; *P* < 0.001; Cohen's *d* = 2.21] and nearly for PO [*t*_(18)_ = 3.03; *P* = 0.007; Cohen's *d* = 1.36], whereas mean PO in S5 was higher in NoF for PO_rel_ [*t*_(18)_ = −3.36; *P* = 0.003; Cohen's *d* = 1.50].

Table 2 provides an overview of the change-segments for both PO and PO_rel_ per group. A group by segment interaction effect was found for both PO [*F*_(3.17)_ = 8.14, *P* < 0.001] and PO_rel_ [*F*_(2.93)_ = 7.81; *P* < 0.001]. *Post-hoc* comparisons revealed that the mean change in PO was higher in FF, compared to NoF, for both PO [*t*_(12.14)_ = 6.08; *P* < 0.001; Cohen's *d* = 2.72] and PO_rel_ in CS9 [*t*_(12.95)_ = 6.06; *P* < 0.001; Cohen's *d* = 2.71].

**Table 2:** **Difference between neighboring 10%-segments within groups (i.e., change-segments)**.

	**PO [W/kg]^b^**			**PO_rel_ [–]^c^**		
**Change-segment**	**FF^a^**		**NoF^a^**	**FF**		**NoF**
CS1^e^ (= S2–S1^d^)	−0.100 (0.277)		−0.011 (0.283)	−0.031 (0.082)		−0.003 (0.083)
CS2 (= S3–S2)	−0.070 (0.056)		−0.043 (0.168)	−0.020 (0.017)		−0.013 (0.049)
CS3 (= S4–S3)	−0.049 (0.120)		−0.007 (0.101)	−0.011 (0.031)		−0.003 (0.032)
CS4 (= S5–S4)	−0.079 (0.147)		0.082 (0.161)	−0.019 (0.037)		0.023 (0.047)
CS5 (= S6–S5)	−0.025 (0.070)		−0.064 (0.135)	−0.008 (0.019)		−0.020 (0.040)
CS6 (= S7–S6)	−0.051 (0.149)		−0.071 (0.080)	−0.009 (0.046)		−0.022 (0.025)
CS7 (= S8–S7)	−0.034 (0.117)		−0.052 (0.111)	0.008 (0.027)		−0.016 (0.033)
CS8 (= S9–S8)	−0.004 (0.084)		−0.109 (0.126)	−0.002 (0.024)		−0.032 (0.038)
CS9 (= S10–S9)	0.449 (0.238)	[^*^]	−0.048 (0.101)	0.130 (0.068)	[§]	−0.014 (0.032)

a FF, Full Feedback control-group (n = 10); NoF; No Feedback experimental-group (n = 10);

b PO, Mean of participants' power output (PO) divided by their body mass;

c PO_rel_, Mean of participants' PO divided by their mean PO over the trial;

d S1–10: 10%-segments;

e *CS1–9: difference between neighboring 10%-segments*.

Found significant differences within the post-hoc Bonferroni corrected independent t-tests for PO and PO_rel_ are marked by, respectively, ^*^ and §.

The segment analysis indicates that the PO-distribution of the groups differed from each other. Most evident is the spurt at the end of the trial of FF, which was not incorporated by NoF. In contrast, NoF increased their PO halfway through the trial and FF did not.

### Perceived exertion

**Figure 3:**
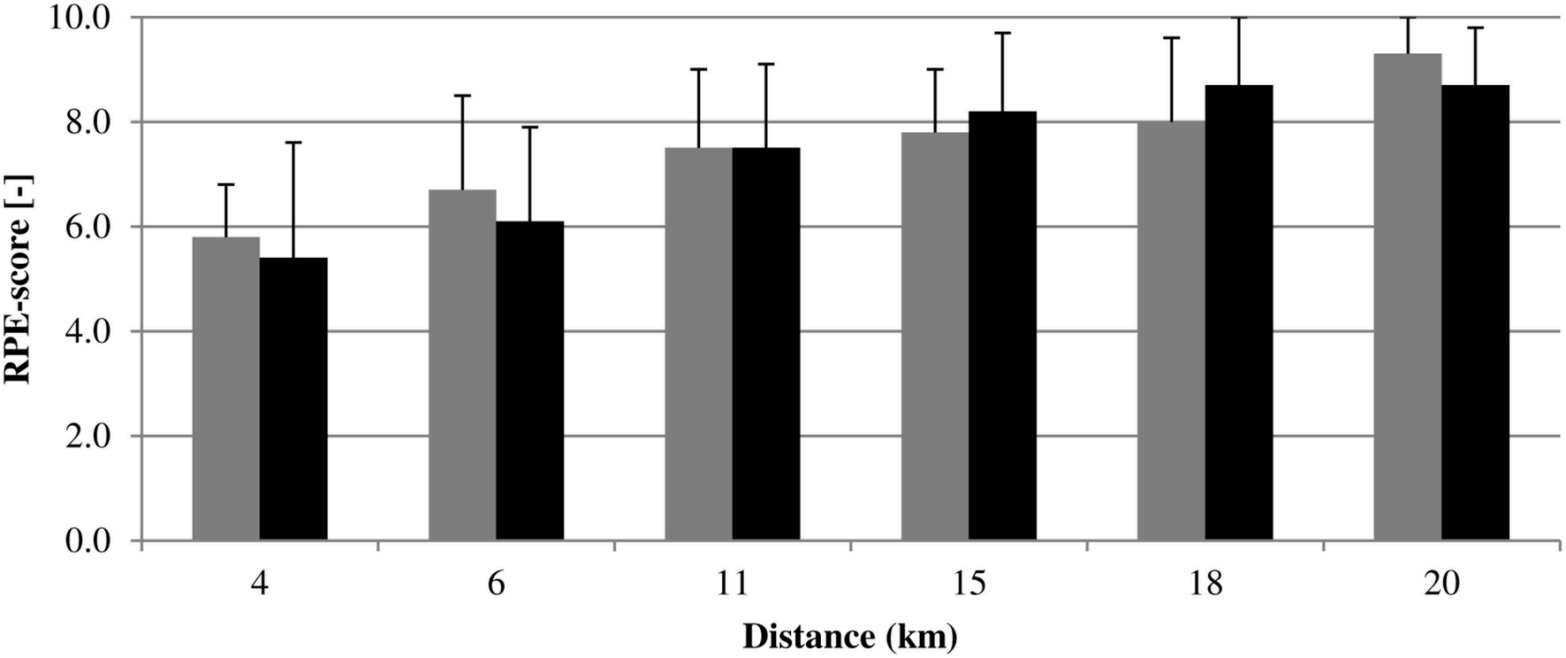
**Comparison of RPE-characteristics [Mean (*SD*)] between groups (*n* = 10 per group) for several moments during the trial**. Gray bars, Full Feedback group; Black bars, No Feedback group; Distance (km), Completed distance (km) within the trial at which the RPE was asked, in which was taken into account that within each 4-km block the RPE was asked at least once. No differences were found.

No differences in perceived exertion scores were found (see Figure 3).

## Discussion

The main aim of the current study was to examine the effects of an absence of task-related feedback on effort distribution and performance in experienced endurance athletes. To do so, pacing and performance during a 20-km cycling time trial of a group that could not consult task-related feedback (NoF) were compared with a group for whom task-related feedback was provided during the entire trial (FF). The results show no spurt at the end of the trial of NoF, whereas FF incorporated an end spurt. Notwithstanding this and other differences in pacing strategy between groups, no difference in overall performance between groups was found. This supports our hypothesis to find no inferior performance in NoF compared to FF. This finding suggests that in middle distance exercise, experienced athletes do not need task-related feedback from external devices to successfully complete a task of which the demands are known. However, the difference in pacing behavior visible toward the end of the race indicates that task-related feedback influences certain aspects of decision-making regarding how and when to invest the available energy over the race.

The lack of performance differences between groups contrasts with the suggestion that cautiousness and a slower speed of completion—designed to reduce errors (e.g., premature exhaustion)—is typically observed in performing motor tasks someone is unfamiliar with (Foster et al., 2009). The PO of NoF was usually at or below the 70%^*^PPO-boundary, whereas FF usually exercised above or at the boundary. Although this finding could suggest that NoF might have included some cautiousness within their pacing strategy, between group analyses of overall performance, PO-segments and RPE did not indicate an obvious structural conservativeness in NoF's pacing strategy compared to FF.

### Performance

A study that compared the performances between groups that did or did not receive distance feedback during multiple 4-km cycling time trials found a better initial trial performance within the group that received distance feedback (Mauger et al., 2009). However, in our study feedback-blinded participants had prior knowledge of the demands (i.e., distance to be covered) of the trial. It has been argued that experience developed during previous (training) bouts reinforces interoceptive sensitivity (Baron et al., 2011). Our participants were experienced in performing exercise bouts of different intensities and duration, and in different environmental circumstances, which makes it possible that they have gained an experience-based awareness of the effort they are able to sustain for endurance trials with different demands (Foster et al., 2004; Hettinga et al., 2006). The absence of feedback-devices meant that our NoF-participants were solely reliant on their own resources (i.e., perceived bodily exertion and prior time trial experience) and prior knowledge of the task demands while distributing their effort over the trial. With this in mind, together with the fact that no performance differences were found between groups, it can be suggested that prior knowledge of task demands together with reliance on bodily information is sufficient for experienced athletes to come to comparable time trial performances when receiving full feedback.

### Effort distribution and perceived exertion

The within-group analysis of power distribution indicates that FF demonstrated a fairly intensive initial phase, followed by a moderate steady middle part, and finishing with an end spurt. Such a parabolic-shaped (i.e., U- or J-shape) strategy is often observed in endurance exercise (Edwards and Polman, 2012). In contrast, NoF showed limited variability in PO within their trial. Moreover, PO- and relative PO-changes differed between groups during the end phase. No PO-change in NoF during the last 10% of the trial was demonstrated, compared to the penultimate 10%, whereas a significant PO-increase in FF during the last 10% was shown. An important implication is that different pacing strategies emerge depending on sources of information available to experienced athletes. Future studies should focus on addressing which information is of importance at what segment of the race, for example by studying gaze behavior and introducing or retracting sources of information during the race (Boya and Micklewright, 2016).

With regard to the end phase; it has been argued that athletes often utilize their remaining energetic reserves—maintained in order to avoid premature exhaustion—in a spurt when they believe they are close to the endpoint of the task (Catalano, 1973; De Koning et al., 2011). The absence of instantaneous task-related feedback made that NoF, in contrast to FF, never had explicit certainty about the remaining distance to be covered, which could have been a considerable interference with determining the moment at which they could exploit their energy reserves. This, in turn, might have prevented them from appealing to their remaining energetic reserves, even though the end phase of the trial was reached. If this were the case, the absence of explicit endpoint knowledge would induce conservativeness during the end phase and hinder maximizing performance. Such a conservative end phase should have led to finishing less exerted compared to finishing with an end spurt. However, this was not supported by our RPE data. Future studies are needed to further explore what will happen when for example introducing endpoint information in the last phase of the race, or what will be the effect of an opponent. In 4-km time trials with known end-point, athletes adapt their strategies to the behavior of their opponent (Konings et al., 2016a). Is this also the case in open-loop exercise?

Taking into account the absence of overall performance- and RPE-differences between groups, together with the limited varied PO-distribution of NoF, it could be suggested that NoF decided to pursue a pacing strategy that enabled personal goal achievement without the incorporation of an end spurt. This pre-planned pacing strategy would be in anticipation to the prior knowledge of the task demands and the knowledge that they would never have explicit certainty of reaching the point after which they could exploit their energy reserves in a spurt. This reasoning fits with recent pacing ideas that decision-making in pacing is based on instantaneous bodily and environmental information, as well as in anticipation to factors such as knowledge of the likely demands of the remaining exercise bout (e.g., certainty about the endpoint and duration) and personal goals (Smits et al., 2014); and pre-planning a pacing strategy using an appropriate situation-specific strategy may be a useful way to distribute effort and optimize performance for that event (Edwards and Polman, 2012).

Within the overall pacing strategy of NoF, characteristics can be recognized from a combination of an evenly paced (i.e., steady PO) and all-out paced (i.e., attempting to maintain a challenging PO for the duration of the bout) strategy. If this is the case, participants in NoF possibly pursued a particular relatively steady but challenging pace they expected to be sustainable for their estimated durations of the trial (possibly based on their experience-based effort-awareness (Hettinga et al., 2006) and including a certain safety margin) and provided a performance that can compete with performances in familiar circumstances as well. The aim of an all-out strategy is to maintain a challenging PO for the duration of the bout, but practical observations suggest PO will deteriorate (Edwards and Polman, 2012); as can also be observed during the end phase of the overall PO-distribution of NoF. Keeping a challenging pace, in turn, should eventually have elicited a considerable perceived exertion in NoF, which can explain why NoF's final RPE does not significantly differ from that of the end sprinting FF-group.

### Pacing and task-related feedback

We examined how the absence of task-related feedback affected time trial execution in experienced athletes, in which the effects of the absence of distance feedback eventually seemed to be most affecting in strategy selection. However, we do not exclude that other task-related feedback could also have been integrated into the decision-making in pacing in FF. Recent research with eye-tracking measurements (Boya et al., 2015) has demonstrated that experienced cyclists who could consult speed-, distance-, PO-, cadence-, HR-, and time-feedback mainly directed their gaze to speed and distance information during their trials. Moreover, it has been suggested that cyclists may choose to pace themselves according to speed feedback if their experience supports this as a successful strategy (Micklewright et al., 2010). Our results further elaborate on the idea that an experience-based awareness of the effort one is able to sustain for different durations of exercise seems robust in time trial exercise (Hulleman et al., 2007). The current study provides a first indication that task-related feedback on external devices, including speed feedback, seems not essential for experienced athletes to come to a comparable endurance performance. This further confirms that interpreting and acting upon bodily information is important in pacing (Smits et al., 2014) and hence recommends exercisers of all levels to pay (more) attention to developing familiarity with self-monitoring (i.e., interpret) and self-regulation (i.e., act) in improving their pacing skills. Also, our results could act as an entry point for reconsidering the way in which task-related feedback on external devices should be used during exercise tasks.

Finally, our results indicate that the consultability of distance feedback (i.e., possibility to gain precise endpoint knowledge) influences effort distribution; which was most obvious during the end phase of the trial. Exercising some cautiousness and (consequently) making situation-based (pre-planned) adjustments to the pacing strategy were proposed as possible consequences of the absence of distance feedback, but our results are not fully conclusive about this. It has already been demonstrated that fit participants with limited specific endurance sports experience were cautious during initial trials (Foster et al., 2009). During later trials, they made adjustments in their strategy and progressively increased effort as they became confident that the time trial could be completed with a particular strategy without negative consequences. Future research with multiple endurance trials should reveal whether such a learning-effect will also occur within experienced feedback-blinded athletes. Lastly, in exercisers' natural (competitive) environment, properties such as optic flow (Parry et al., 2012) as well as the presence of opponents (Konings et al., 2016b) have been shown to be of influence on performance and decision-making in pacing. Such properties were not incorporated in the present experimental set-up as yet. Future research should thus also be arranged with experimental conditions that are representative of the exercisers' natural environments (Smits et al., 2014) to explore the impact of environmental properties on exercise performance and pacing while external feedback devices are present or not.

## Author contributions

FH and BS designed the study protocol, BS and RP conducted the initial data analysis. All authors contributed to interpretation and conception of the work as well as further development of the proposed ideas. BS wrote a first draft of the work. All authors were involved in further drafting, editing, and final approval of the manuscript.

### Conflict of interest statement

The authors declare that the research was conducted in the absence of any commercial or financial relationships that could be construed as a potential conflict of interest.
